# Forecasting Large-Scale Habitat Suitability of European Bustards under Climate Change: The Role of Environmental and Geographic Variables

**DOI:** 10.1371/journal.pone.0149810

**Published:** 2016-03-03

**Authors:** Alba Estrada, M. Paula Delgado, Beatriz Arroyo, Juan Traba, Manuel B. Morales

**Affiliations:** 1 CIBIO/InBIO, Universidade de Évora, Évora, Portugal; 2 Instituto de Investigación en Recursos Cinegéticos—IREC (CSIC-UCLM-JCCM), Ciudad Real, Spain; 3 Terrestrial Ecology Group (TEG), Department of Ecology, Universidad Autónoma de Madrid, Madrid, Spain; Ecologie, Systématique & Evolution, FRANCE

## Abstract

We modelled the distribution of two vulnerable steppe birds, *Otis tarda* and *Tetrax tetrax*, in the Western Palearctic and projected their suitability up to the year 2080. We performed two types of models for each species: one that included environmental and geographic variables (*space-included* model) and a second one that only included environmental variables (*space-excluded* model). Our assumption was that ignoring geographic variables in the modelling procedure may result in inaccurate forecasting of species distributions. On the other hand, the inclusion of geographic variables may generate an artificial constraint on future projections. Our results show that *space-included* models performed better than *space-excluded* models. While distribution of suitable areas for *T*. *tetrax* in the future was approximately the same as at present in the *space-included* model, the *space-excluded* model predicted a pronounced geographic change of suitable areas for this species. In the case of *O*. *tarda*, the *space-included* model showed that many areas of current presence shifted to low or medium suitability in the future, whereas a northward expansion of intermediate suitable areas was predicted by the *space-excluded* one. According to the best models, current distribution of these species can restrict future distribution, probably due to dispersal constraints and site fidelity. Species ranges would be expected to shift gradually over the studied time period and, therefore, we consider it unlikely that most of the current distribution of these species in southern Europe will disappear in less than one hundred years. Therefore, populations currently occupying suitable areas should be a priority for conservation policies. Our results also show that climate-only models may have low explanatory power, and could benefit from adjustments using information on other environmental variables and biological traits; if the latter are not available, including the geographic predictor may improve the reliability of predicted results.

## Introduction

Climate change is considered one of the major drivers of changes in species distribution [1,2]. Studies addressing the effect of climate change on biodiversity have been performed at different spatial extents and resolutions, varying from local or provincial studies [3,4] to national, continental or global ones [5–7]. Normally, studies performed at continental or global scales include many species in an attempt to obtain general patterns of the possible effect of climate on biodiversity [7,8]. While these approaches allow the identification of global risks and concerns, it is also necessary to examine the effect that climate change may have on particular endangered species in order to guide specific conservation practices [9,10].

Additionally, the majority of biogeographical large-scale studies only include climatic variables in the modelling procedure [6,11]. However, it is unlikely that a species’ large-scale distribution will depend only on climate, as environmental predictors such as topography or land use typically affect species distributions [10,12,13]. In addition, variables describing the spatial structuring of the species allow the inference of the possible roles of population dynamics, dispersal capacities, and historical events on species distributions [14]. Some species distribution models (SDMs) are Generalized Linear Models (GLMs) that theoretically assume independence between cells, i.e., that presences/absences in specific cells are independent of one another. However, organisms are not randomly or uniformly distributed in the natural environment because processes such as growth, reproduction, and mortality, which create the observed distributions of organisms, generate spatial autocorrelation in the data (i.e. lack of independence due to geographic proximity). Therefore, SDMs must include realistic assumptions about the spatial structuring of communities [15] to avoid the misinterpretation of the ecological mechanisms that explain species ranges [10,16,17]. This is particularly important when forecasting the distribution of declining species associated with specific habitats or topographic characteristics, because conservation guidelines based on inaccurate forecasting may be particularly detrimental. Thus, the estimation of the effect of future climate on the distribution of declining species should be performed by combining climate with environmental predictors such as topography or land use, and taking spatial structuring into account.

The little bustard *Tetrax tetrax* and the great bustard *Otis tarda* are two steppe-bird species categorized as vulnerable at a global scale [18]. Both species are distributed across the Western Palearctic, but their population strongholds in that area are concentrated in the Iberian Peninsula [19,20]. As with other steppe birds, they preferentially occupy open flat areas with low vegetation [21], which in Europe mainly correspond to cereal croplands and pastures, where they often occur sympatrically. They share landscape-scale breeding habitat preferences (flat, open grassland and extensive cereal farmland) [22,23] and food requirements (they are both mainly herbivorous, although the young rely heavily on insects during their first weeks of life) [24,25], and they differ principally in their selection of agricultural habitat types and vegetation structure [23,26,27].

There are studies addressing the effect of environmental variables on these two steppe-bird species. At local or regional spatial extents (e.g. natural reserves or provinces), different authors have shown that both climate and habitat variables have an influence on the abundance or breeding success of these species [28–31]. At wider continental extents and resolutions (e.g. 50 km x 50 km UTM cells in Europe), other studies have focused on the effect that climate may have on their distributions [32–34]. However, no study has yet attempted to model the continental distribution of these two endangered steppe-bird species under climate change scenarios, incorporating non-climatic environmental variables such as land use and topography on forecasted suitability, as well as accounting for spatial structuring.

We aimed to evaluate the effect of climate, land use, topography and geographic variables on the distribution of both species, and project their future suitability under different climate change scenarios. To project suitability we followed two different approaches: one that accounts for environmental and geographic variables, and another that only takes environmental variables into account. Our assumption was that species’ distributions may be spatially constrained beyond environmental variables and therefore, future suitability should account for the geographic configuration of the species. On the other hand, the inclusion of geographic variables may represent an artificial constraint on future projections, as the spatial structure in bird data may also change in the future, in interaction with climate. Therefore, here we address the following question: Are geographic variables informative and thus, should they be included in SDMs aimed to detect the effect of climate change on species distributions? We discuss the implications of our results for the conservation of both steppe birds in the Western Palearctic.

## Materials and Methods

### Bustard distribution data and study area

**Fig 1:**
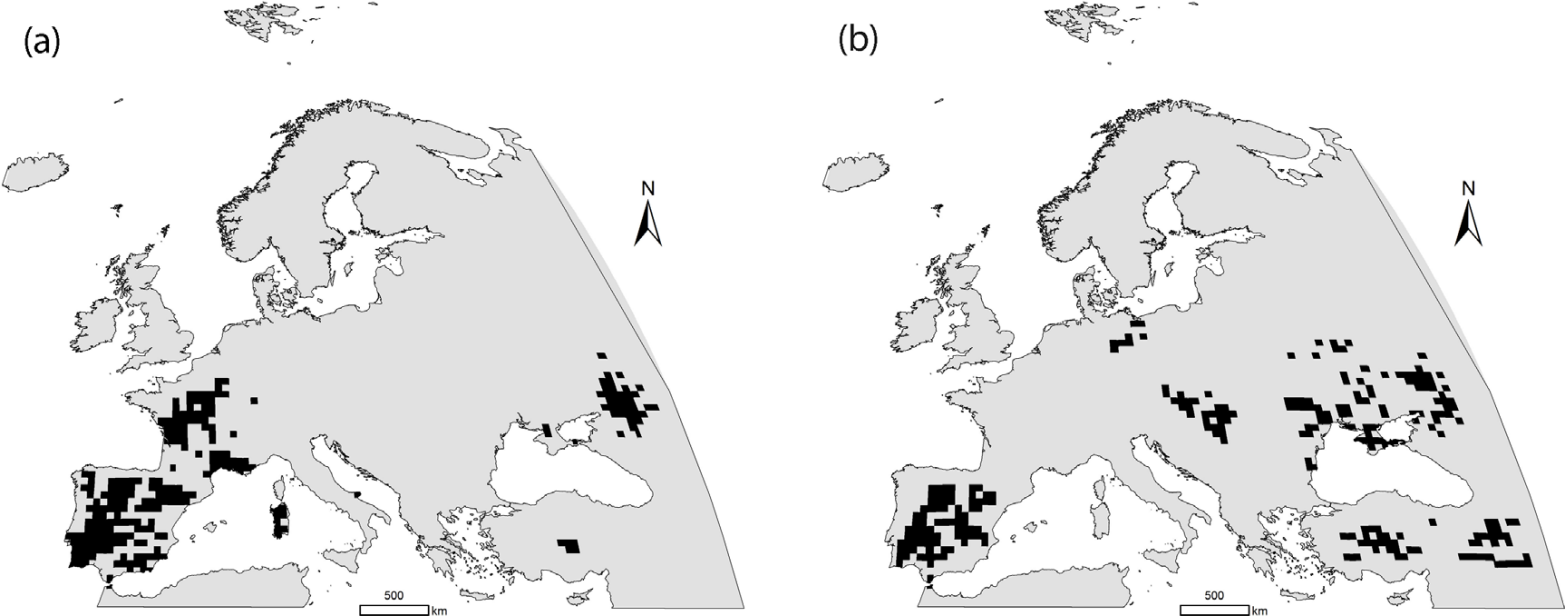
Distribution of the little (a) and the great bustard (b) in the study area.

We used breeding presence/absence data for both species in a grid of 50 km x 50 km UTM cells (n = 4532) according to the European Atlas of Breeding Birds [35]. Data were obtained from the European Bird Census Council (EBCC) and have the form of presence or absence in each UTM cell. In order to complete their ranges in the Western Palearctic, data for the two species in Turkey [36] and Morocco [37,38] were added to the database (total number of presences = 250 and 260 for the little and the great bustard, respectively, Fig 1). The distribution data used for both species correspond to the widest area for which they were available: both species also occur in parts of Asia [21], but breeding data from that continent are either unavailable or unreliable, and hence not comparable with the European atlas data or the cited reports for Morocco and Turkey. In this study area and at this resolution we can consider that cells with no presence are true absences. The study area thus comprises the majority of Europe, North Africa and Southwest Asia, which covers the two species’ entire ranges in the Western Palearctic and coincides with the study area used by Delgado et al. [33] in a previous study on these species’ climatic niche (Fig 1).

### Predictor variables

Regarding climatic variables, raw temperature and precipitation data were extracted from WorldClim (http://www.worldclim.org/) according to the Climgen Statistical Downscaling for the ‘current’ period 1961–1990 and for the future periods 2050 and 2080, the latter periods according to the emission scenario A1B in three different general circulation models (GCMs): CGCM31, ECHAM5 and HADCM3. We selected three GCMs and one emission scenario because discrepancies in predictions are higher between different GCMs than between different emission scenarios in a specific GCM [5,39]. Additionally, the inclusion of different emission scenarios would have likely only produced differences in the magnitude of change rather than in the direction of change [40]. We selected the A1B emission scenario because it is at an intermediate position between low and high sustainable development. The A1B scenario describes a future world of very rapid economic growth, global population that peaks in mid-century and declines thereafter, and the rapid introduction of new and more efficient technologies. This scenario assumes a balance across fossil and non-fossil energy sources, not relying too heavily on one particular energy source, on the assumption that similar improvement rates apply to all energy supply and end-use technologies. CO_2_ emissions in the A1B scenario are higher than in B1 and B2 scenarios [41].

For each GCM we calculated three bioclimatic variables that had been previously shown to have an effect on the distribution of both species [33]. These were cumulative annual rainfall, as a general description of water availability; temperature range between July and January, which describes the thermal gradient from oceanic to continental climate; and the mean temperature during the reproductive period for both species, i.e. between April and July (Table 1). We considered the possibility of unimodal responses of the species to these climatic variables by including their quadratic terms.

**Table 1:** Variables, and their associated predictor sets, used in the modelling procedure.

Predictor set	Variable	Code
Climate	Cumulative annual rainfall (mm)^1^	Pannual
	Temperature range (July–January) (°C)^1^	Range
	Mean temperature April–July (°C)^1^	Tapr–jul
Land use	% Dry crops and pastures^2^	DCP
	Human population density (number of inhabitants/km^2^)^3^	HPd
Topography	Slope (°) ^(derived from^ ^4^^)^	Slope
Geographic location	Trend surface variable	Geog

Sources:

^1^ WorldClim (http://www.worldclim.org/)

^2^ USGS Land Cover (http://edc2.usgs.gov/glcc/glcc.php)

^3^ ORNL [43]

^4^ GLOBE et al. [42].

As both species appear in flat open areas and are associated with dry crops [29], the following explanatory variables were also considered (Table 1): the mean slope of the UTM cell (derived from GLOBE et al. [42]) and the percentage of dry crops and pasturelands in each cell (obtained from the USGS Land Cover, http://edc2.usgs.gov/glcc/glcc.php). Additionally, we included the mean value of human population density (obtained from ORNL [43]), as most steppe-bird species avoid areas of dense human populations [22]. Predictor variables were processed in ArcGIS 10 [44].

Finally, we considered a geographic descriptor to control for spatial autocorrelation and to test the existence of pure spatial range constraints, such as historical or spatial ecological dynamics [45]. This descriptor was defined for each species following the ‘‘trend surface approach” [15]. This approach allows accounting for the effect of subjacent spatial structures that are not captured by the environmental and human predictors considered [46]. For this, a series of combinations of longitude (X) and latitude (Y) of the central point of each grid cell (X, Y, X^2^, Y^2^, X^3^, Y^3^, XY, X^2^Y, XY^2^), calculated in ArcGIS, were entered in a backward stepwise logistic regression [15]. The ‘‘trend surface variable” was then considered to be the logit resulting from the geographic model, i.e., the linear combination of geographic variables resulting from the logistic regression [14,47]. The dataset is available at http://hdl.handle.net/10261/128941.

### Modelling method

We applied the favourability function [48] to the distribution of both species following the modelling approach detailed below. All analyses were performed in IBM SPSS Statistics 21 [49].

Firstly, we grouped the variables into four predictor sets, namely geographic location, topography, land use, and climate (Table 1). For each species and predictor set, we controlled the type I error by evaluating the false discovery rate (FDR [50]), accepting the variables that were significant under a FDR of q < 0.05 within the predictor set. Secondly, we calculated a model for each predictor set independently (i.e. predictor-set model) performing forward-backward stepwise logistic regression on the variables that were retained in the FDR test. Finally, we obtained combined models for each species by performing forward-backward stepwise selection on all the variables that were included in each predictor-set model [10]. Inclusion or exclusion of variables in the stepwise selection of predictor-set and combined models was based on significance testing. We selected logistic regression and a stepwise approach because we were interested in knowing which variables explained broad-scale distribution of the species (i.e., those variables that first enter to form part of the model) and which of them act on fine-scale distribution patterns (i.e., those entering last) [10]. Additionally, the stepwise approach has recently been described as one of the best methods to combine SDMs based on different sets of predictors [14]. We performed two types of combined models for each species: one that included environmental and spatial variables (climate, topography, land use and the geographic predictor), which we designated as the *space-included* model; and a second one that included only environmental variables (climate, topography and land use), which we designated as the *space-excluded* model (see the rationale for performing two types of models at the end of the Introduction). Combined models were trained on a 70% random sample of the original data and predictive accuracy was tested on the remaining 30% [51,52]. We obtained suitable areas for each species after applying the favourability function [48] to the output of the combined models. We selected this function because, in contrast to probability values derived from logistic regression, favourability values are not affected by the ratio presences/absences of the species and reflect only the conditions that are environmentally suitable for them [53]. Therefore, favourability models are directly comparable between species and their use is recommended in predictive models like the ones performed in the present study [48].

Favourability can be obtained from logistic regression probabilities estimated using GLMs as follows [48]:

$$F=\frac{\frac{P}{\left(1-P\right)}}{\frac{n_{1}}{n_{0}}+\frac{P}{\left(1-P\right)}}$$

where P is the probability value in a cell, n_1_ is the total number of presences and n_0_ is the total number of absences. Favourability values range from 0 to 1. We obtained a favourability value for each species in each 50 km x 50 km UTM cell.

Multicollinearity of all the variables that entered into combined models was checked with the variance inflation factor (VIF). We assessed the discrimination power of the combined models with the validation dataset (30% of data) by estimating the sensitivity (proportion of presences correctly classified), specificity (proportion of absences correctly classified), and their Correct Classification Rate (CCR, proportion of cases correctly classified), using the neutral favourability value of F = 0.5 as the classification threshold. We also calculated the Area Under the Curve (AUC) of the Receiver Operating Characteristic [54–56], which is independent of any favourability threshold [57]. We calculated the percentage of deviance explained (i.e., how much variation in the response is explained by the model) according to Nagelkerke’s R^2^. Finally, we compared the parsimony of the combined models using the Akaike Information Criterion (AIC) [58].

We performed a variation partitioning of the combined models for both species in order to determine how much of the variation of the complete model was explained by the pure effect of each predictor set (namely climate, topography, land use and geographic), and which proportion was attributable to their shared effect (i.e., it cannot be attributed to one predictor set or another) due to spatial collinearity [15,46,59,60].

Finally, suitable areas for each species were projected to the future by replacing the current climatic values in the combined favourability models with those predicted for the periods 2050 and 2080. We forecasted the two types of combined models, i.e., the *space-included* model and the *space-excluded* model.

To ascertain that our results are generalizable for different SDMs, we also modelled the distribution of both species with three other modelling techniques (Boosted regression trees, Random Forest and Multiple Adaptive Regression Splines). Methods, results and projections with these techniques are in S2 Appendix.

## Results

**Fig 2:**
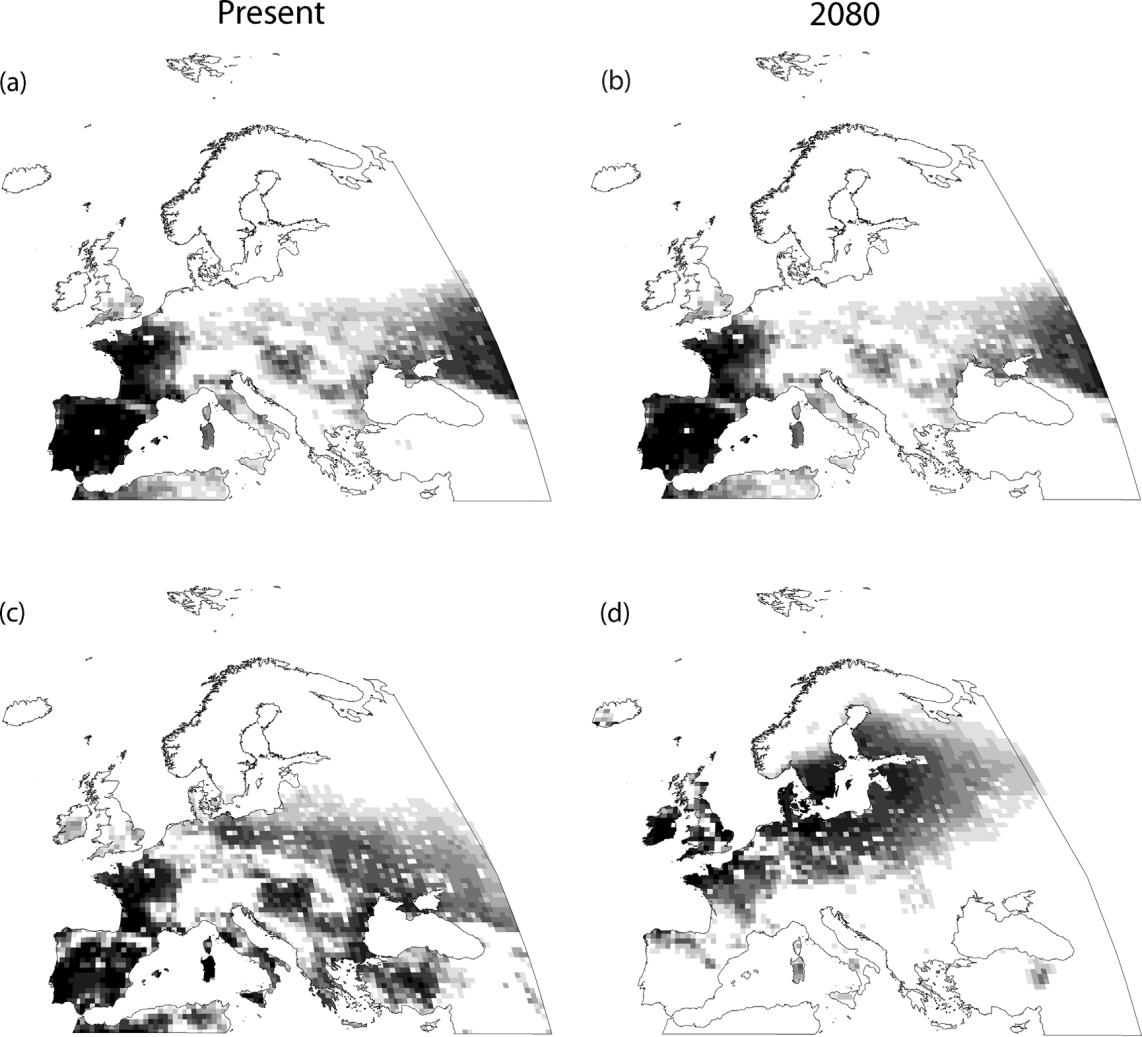
Favourability for the little bustard at present and in 2080. a) Present favourability according to the *space-included* model; b) future favourability in 2080 according to the *space-included* model and the GCM HADCM3; c) present favourability according to the *space-excluded* model; d) future favourability in 2080 according to the *space-excluded* model and the GCM HADCM3. Favourability ranges from zero (white cells) to one (black cells). Classification maps with high, medium and low favourability are represented in Figure A in S1 Appendix.

**Fig 3:**
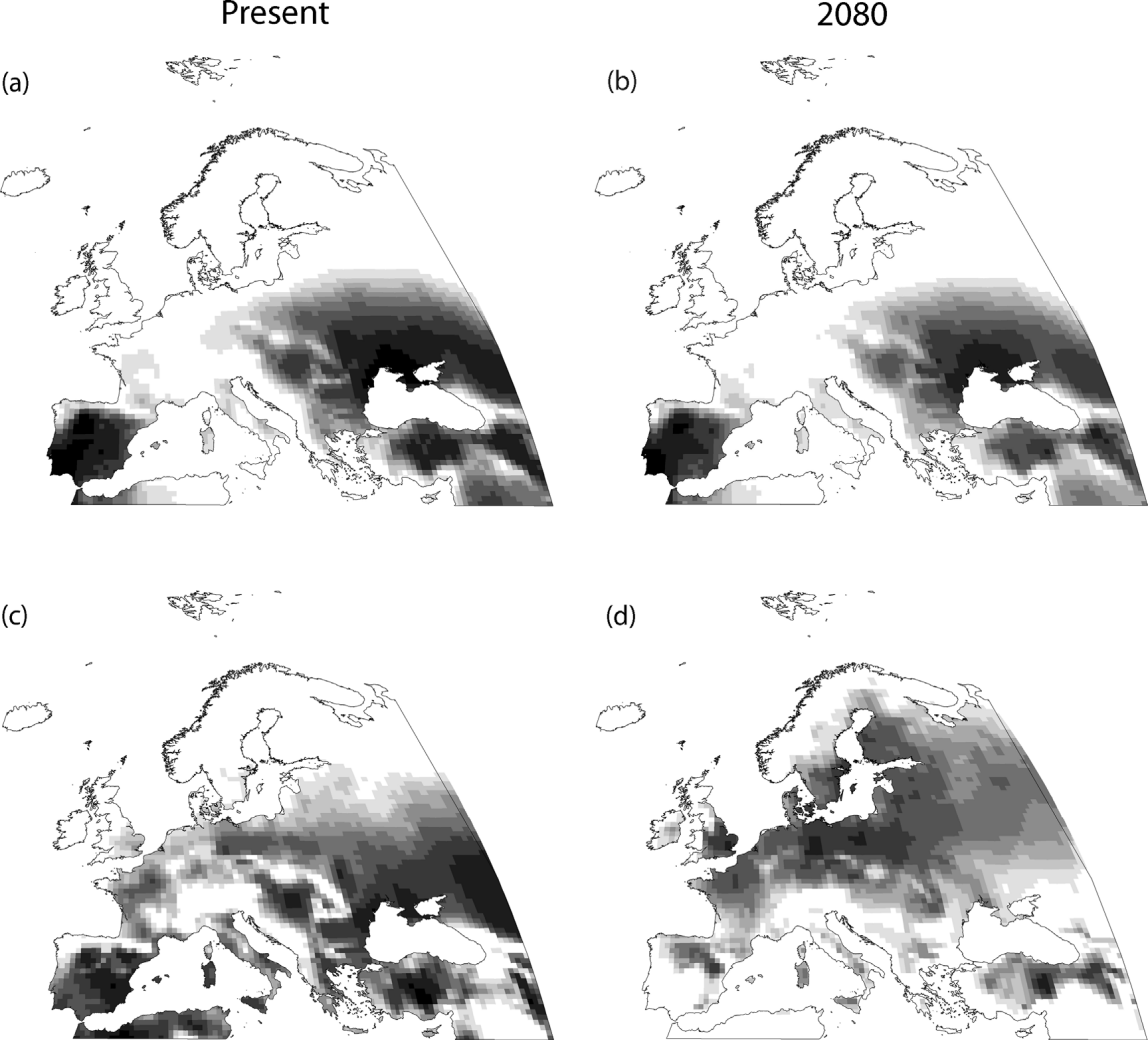
Favourability for the great bustard at present and in 2080. a) Present favourability according to the *space-included* model; b) future favourability in 2080 according to the *space-included* model and the GCM HADCM3; c) present favourability according to the *space-excluded* model; d) future favourability in 2080 according to the *space-excluded* model and the GCM HADCM3. Favourability ranges from zero (white cells) to one (black cells). Classification maps with high, medium and low favourability are represented in Figure B in S1 Appendix.

**Fig 4:**
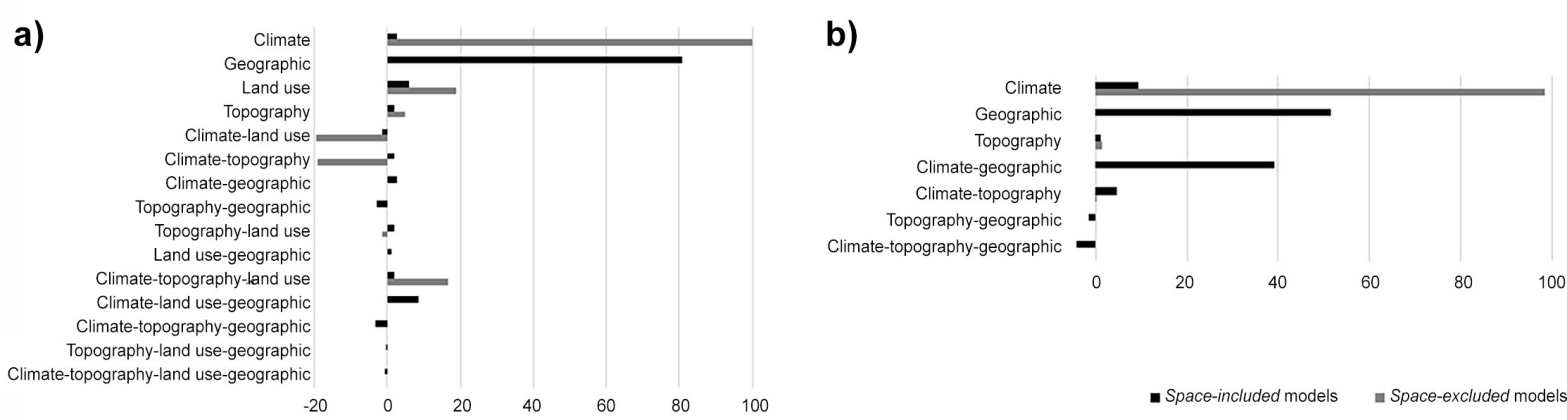
Variation partitioning of predictor sets explaining favourability for little bustard (a) and great bustard (b). Values shown in the bars are the percentages of variation of the models explained exclusively by climate, the geographic predictor, land use, topography, and by the combined effect of these predictor sets. Note that each model can be formed by different numbers of predictor sets (see Tables 1 and 3).

Variables selected in the predictor-set models are shown in Table 2. Except human population density for the great bustard, and the quadratic terms of annual precipitation and temperature range for both species, all other variables are potential candidates to be included in the combined models. Modelled current favourable areas for each species in the Western Palearctic are shown in Figs 2 and 3. The variables that affect their breeding distributions at this scale are shown in Table 3, while the results of the variation partitioning of the combined models for each species are represented in Fig 4. Multicollinearity between explanatory variables entered into combined models was low: the maximum VIF value was 2.65.

**Table 2:** Variables selected in the predictor-set models.

Predictor set	Variable	Little bustard	Great bustard
Climate	Pannual	x	x
	Pannual^2^		
	Range	x	x
	Range^2^		
	Tapr–jul	x	x
	Tapr–jul^2^	x	x
Land use	DCP	x	x
	HPd	x	
Topography	Slope	x	x
Geographic location	Geog	x	x

Variable codes as in Table 1: Pannual: cumulative annual rainfall, Pannual^2^: quadratic term of Pannual, Range: temperature range (July–January), Range^2^: quadratic term of Range, Tapr-jul: mean temperature April–July, Tapr-jul^2^: quadratic term of Tapr-jul, DCP: % dry crops and pastures, HPd: human population density, Geog: trend surface variable, see Methods.

**Table 3:** Combined models for Little (LB) and Great Bustard (GB) current favourability and their evaluation metrics.

	LB space-included				LB space-excluded				GB space-included				GB space-excluded			
	β	SE	Sig	Order	β	SE	Sig	Order	β	SE	Sig	Order	β	SE	Sig	Order
Pannual	-0.0028	0.0006	***	3	-0.002	0.001	***	5	-0.0062	0.0008	***	3	-0.005	0.001	***	3
Range	-0.15	0.03	***	5	-0.35	0.03	***	2					-0.05	0.02	*	5
Tapr–jul					5.85	0.67	***	1	-0.17	0.04	***	1	3.33	0.47	***	1
Tapr–jul^2^					-0.18	0.02	***	3					-0.1	0.014	***	2
DCP	0.021	0.004	***	2												
HPd	-0.009	0.002	***	6	-0.020	0.003	***	6								
Slope	-0.21	0.05	***	4	-0.24	0.04	***	4	-0.11	0.03	**	4	-0.086	0.034	*	4
Geog	1.08	0.09	***	1					1.20	0.10	***	2				
Intercept	4.75	0.89	*		-37.78	5.16	***		6.25	0.90	***		-22.46	3.77	***	
DE	55%				39%				37%				26%			
AUC	0.964				0.912				0.903				0.869			
Sensitivity	0.973				0.827				0.885				0.923			
Specificity	0.868				0.787				0.757				0.676			
CCR	0.874				0.789				0.764				0.69			
AIC	690.77				912.07				956.37				1099.2			

We present two models for each species, i.e. including and excluding the geographic predictor (space-included and space-excluded, respectively). β: coefficients; SE: standard errors; Sig: significance:

***<0.001

**<0.01

*<0.05, ns: not significant; Order: order of entrance in the model. Variable codes as in Table 1: Pannual: cumulative annual rainfall, Range: temperature range (July–January), Tapr-jul: mean temperature April–July, Tapr-jul^2^: quadratic term of Tapr-jul, DCP: % dry crops and pastures, HPd: human population density, Geog: trend surface variable, see Methods. DE: deviance explained; CCR: correct classification rate.

According to model coefficients, the most favourable areas for the little bustard were those with lower human population density, annual precipitation and slope, for both the *space-included* and the *space-excluded* combined models. Additionally, the species avoided areas with a marked continental climate, as the temperature range was inversely related to its favourability (Table 3). The proportion of dry crops and pastures was positively related to this species’ distribution, although this variable only entered in the *space-included* model. On the other hand, a unimodal relationship with temperature during the reproductive period was observed in the *space-excluded* model. In the former model, the pure influence of the geographic predictor explained most of the variation (80.64%, Fig 4A), while the pure influence of land use, slope and climatic variables accounted for much smaller proportions (6.09%, 1.97% and 2.48%, respectively). The proportion of variation explained by the overlaid effect of two or more predictor sets was very small in all cases (and smaller than the pure effects), except in the case of the combination of climate, land use and the geographic predictor with 8.36% (Fig 4A). On the contrary, the model that excluded the geographic predictor was explained mainly by the pure effect of climate (99.97%). In this model, the joint effect of climate with both land use and topography was negative indicating that these predictor sets have opposite geographic effects on the explained favourability [61], i.e., that their relationship is mostly suppressive and not additive [46]. The reason why the combined effect of climate and the geographic predictor was so low for the little bustard in the *space-included* model (Fig 4A) can be related to the type of climatic variables that formed part of the model. In the *space-excluded* model the temperature of the reproductive period was included in the model (Table 3). Contrarily, this climatic variable was not selected in the *space-included* model. Therefore, it seems that the climatic variable that is spatially structured for this species is the temperature of the reproductive period. However, in the *space-included* model the effect of the geographic location was higher and sufficient to capture the effect of temperature on the breeding distribution of this species. Both combined models for this species presented an outstanding discrimination capacity according to the thresholds of AUC proposed by Hosmer and Lemeshow [57], although the AUC of the *space-excluded* model was slightly lower than that of the *space-included* model (Table 3). The inclusion of the geographic pattern increased the percentage of deviance explained by around 20% in the little bustard’s models (Table 3) and all other discrimination metrics were better in the *space-included* model than in the *space-excluded* model. AIC values reflect that the *space-included* model was more parsimonious than the *space-excluded* model. Consequently, modelled current favourability was better defined when the geographic pattern was considered (Fig 2A).

Favourability for the great bustard differed whether or not the geographic predictor was included in the model. In the *space-excluded* model, the species showed high favourability in areas with lower slope, precipitation and temperature range, and in areas with intermediate values of temperature during the reproductive period (unimodal response). In the *space-included* model, favourable areas were those with lower slope, annual precipitation and temperature in the reproductive period (Table 3). Thus, the *space-included* model was formed by three predictor sets (i.e., climate, topography and the geographic predictor). The geographic predictor accounted for 51.59% of the variation (Fig 4B) but the overlaid effect of this predictor set with climate was also very important (39.15%), suggesting that climatic influence on species distribution was spatially structured. The variation explained by the pure topographical predictor set was very low (1.13%). On the other hand, only two predictor sets were involved in the *space-excluded* model, the pure effect of climate being the one that explained almost all the proportion of the variation (98.49%), whereas the pure effect of topography and the combined effect of topography and climate explained a very low proportion (1.44% and 0.07% respectively). The *space-included* and the *space-excluded* model presented outstanding and excellent discrimination capacity respectively according to the thresholds of AUC proposed by Hosmer and Lemeshow [57] (Table 3), but the percentage of deviance explained increased by around 10% in the *space-included* model (Table 3). All other discrimination metrics, except the sensitivity, performed better in the *space-included* than in the *space-excluded* model, and AIC values were lower in the *space-included* model. Consequently, the *space-included* model better defined current favourability for the great bustard (Fig 3A).

In stepwise modelling, the first variables entering the models are those explaining broad-scale distribution, whereas those entering last mainly act on fine-scale distribution patterns [10]. Thus, our results show that breeding favourability for the little bustard in the Western Palearctic in the *space-included* model is affected, in the first place, by the geographic location, secondly by land use, and only thirdly by climatic predictors (Table 3). More precisely, the presence of dry crops and pastures was the second most relevant variable explaining the distribution of favourable areas for the little bustard at a large scale, followed by climatic variables (Table 3). When we excluded the geographic pattern, climatic variables were the first to enter the model and the importance of dry crops was diluted (Table 3), but this model had higher AIC and therefore performed worse. In the case of great bustards, land-use variables do not seem to affect breeding distribution at a continental scale (Fig 4B, Table 3). Great bustard large-scale distribution in the *space-included* model was explained by two climatic variables, annual precipitation and temperature during the reproductive period, the latter being the first to enter the model. This variable was also the first one to form part of the *space-excluded* model (Table 3). The sign of the coefficient for this variable was, however, different in both models: the univariate relationship of great bustard distribution with temperature was positive, but this value turned to negative when the geographic predictor and precipitation were included in the model, indicating that the positive relationship was accounted for by these two variables and their residuals have a negative relationship with the temperature of the reproductive period.

In the case of future favourability, we show in Figs 2 and 3 the results for 2080 according to the GCM HADCM3, as patterns were similar for the different GCMs. Favourability maps for 2050 and 2080 in all GCMs are shown in Figs C-F in S1 Appendix. Forecasted favourability was different for models with and without the geographic predictor (Figs 2 and 3). Although current favourability for the little bustard was roughly similar with both types of models, the forecasted future favourability was totally different in both situations. Favourability for this species in the future was approximately the same as in the present period in the model that includes the geographic pattern, while future favourability in the absence of a spatial constraint was located in the north-western part of the study area, suggesting a pronounced change of favourable areas for this species in the future (Fig 2). In the case of the great bustard, future suitability patterns were similar to those modelled with current climatic data in the *space-included* model, although many areas of current presence showed low or medium future favourability. On the other hand, future favourability in the *space-excluded* model showed a northward expansion of intermediate suitability areas (Fig 3).

Very similar patterns are obtained with the three other modelling techniques applied, i.e., although predicted suitable areas are not exactly the same with the different SDMs (as expected), in all cases discrimination capacity is improved by the inclusion of geographic variables in the modelling procedure, and projections suggest a marked change of future suitable areas in the *space-excluded* models (S2 Appendix).

## Discussion

Our study identifies differences in accuracy and projections of SDMs performed with and without the inclusion of spatial structuring. Previous studies have highlighted that ignoring the geographic component in the modelling procedure may result in inaccurate forecasting of species distributions [10,16,17]. In accordance with this, we show that favourability models that take into account the geographic pattern of the species perform better than models that exclude this predictor, and this result is generalizable to other SDM approaches (S2 Appendix).

Our results are in accordance with those of Suárez-Seoane et al. [29] who found that the percentage of dry crops was the most important variable explaining the distribution of the little bustard at a regional extent, while climatic variables were less relevant. Regarding climate, our results show that the little bustard prefers areas with low annual precipitation and low temperature range, consistent with results found by Delgado et al. [33]. Additionally, this species avoids areas with high human population density, an expected result that is in accordance with the avoidance of human disturbance by little bustards in the Iberian Peninsula found by Suárez-Seoane et al. [22].

In the case of great bustards, other authors have found that the presence of cereal fields and the distance to human infrastructures have an effect on local or national distribution, or on selection of nest-sites in this species [22,31]. However, our results show that great bustard distribution patterns at a continental level can be better explained by climate, topographical and geographic variables, also suggesting that the great bustard is probably more generalist in terms of habitat needs than the little bustard, as pointed out by previous works at a local level [23,27].

In the best models for both species, i.e., those that included the geographic predictor, this was, by far, the most relevant in explaining their current breeding distribution of both species at a European scale. This suggests that the distributions of these species at a continental scale are spatially constrained beyond the effect of environmental variables [16]. Here we detail some possible explanations of this result, based on the biology of the two species. First, this result suggests that historical events and subsequent large scale migration/dispersion dynamics (that resulted in pure spatial trends) seem to have played an important role in the current configuration of their distributions [10,45,62]. It also suggests that processes that determine the geographic range of the species and that are not detectable at a continental scale due to the wide spatial resolution, such as conspecific attraction, local availability of adequate habitat, or local extinction and colonization, may define the distribution of the species and how it is going to evolve. In this sense, both species are known to be strongly philopatric and show low natal dispersal, although this behaviour may vary according to factors like the size of the natal population [63,64]. Similarly, both species show strong conspecific attraction mediated by their lekking mating systems [65,66]. Due to its high relevance in explaining the variation of the model, AIC and discrimination capacity, we encourage the use of the geographic location in SDMs, as it can be considered an effective proxy for unmeasured environmental covariates or population processes such as conspecific attraction and dispersal [67]. Not including the geographic location in modelling approaches would imply the conscious omission of relevant processes underlying the distribution of species.

### Future distribution changes

Regarding how climate change will affect favourable areas for the little bustard, our results show that its potential future breeding distribution will be approximately the same as at present if we take the geographic predictor into account. Nevertheless, favourability in the eastern part of its distribution range will slightly decrease in the future. Huntley et al. [68], using only climatic variables, forecasted a reduction in the potential distribution area of the species at the end of the 21st century, with a strong northward shift of the species’ core range, and with most of the current southern localities, including those in its Iberian stronghold, predicted as unsuitable. We have obtained a similar (or even more severe) pattern in the model that excludes the geographic predictor. These latter projections fall on a geographic range quite distant from current distribution, which is highly unlikely to be occupied in 65 years given the mentioned constraints to the species’ dispersal. It is worth noting that projections could be inaccurate if there were some errors in the predictors [40,69]. We could have accounted for this using the uncertainty estimates of the climatic variables [69] but we lack this information for the other environmental variables. In any case, the main results obtained would have not likely changed, since the same potential errors would have been included in both approaches.

Our results in the best model for the little bustard highlight that land use may have an effect as important as climate in the breeding distribution of the species during the 21st century. In our models, we maintained the percentage of dry crops and pastures constant throughout the years assuming that crops producing food are unlikely to diminish in the present century. If they were to decline, that would have a strong impact in little bustard distribution. In this context, Princé et al. [70] showed that appropriate land-use changes may mitigate the negative effect of climate change on farmland bird species in France. Further studies on future distribution and effects of climate change on these globally threatened steppe-bird species should address the interaction between climate and land use in other study areas, e.g. through changes in agricultural practices, which may also have profound impacts on bird populations inhabiting farmland areas.

In the case of great bustards, we forecasted a slight negative effect of climate change on the breeding distribution of this species when the geographic pattern is considered. Some of these most favourable areas in the future are in accordance with those found more suitable for the species by Synes and Osborne [34]. Additionally, we obtained unfavourable areas for the great bustard in central Europe where there has been a dramatic decline of the species’ populations [71]. Thus, the situation may be even worse if climate change predictions are met. However, in the *space-excluded* model, future favourable areas are distributed all over the Western Palearctic, especially in the north, although with intermediate favourability values. The reduction in this species’ distribution forecasted by Huntley et al. [68] was stronger than that obtained in our work, and almost the entire present range was predicted to be no longer suitable by those authors. This may have encouraged reintroduction projects, for instance, for the great bustard in southern England [72]. However, our models show that future favourability for this species will not necessarily be higher in that area (even in the *space-excluded* model favourability has intermediate values), and we believe that, in the long term, it may be more sustainable to adequately manage populations in current high favourability areas than to try to create new ones far away from the cores of current distribution.

### Conclusions and future research

Our results show that commonly used climate-only models may have low explanatory power, and are likely missing many important biological and environmental factors. Moreover, projections of *space-excluded* models are mainly driven by climatic variables, and this can be an artefact of the spatial structuring of the species’ distribution relative to the climatic variables. Therefore, the importance of climatic variables is most likely inflated in *space-excluded* models, which is a potential drawback for many SDMs, particularly for those commonly used that only consider climate in the modelling procedure. This effect may be accentuated in future predictions, where favourable areas track climatic gradients. Thus, we encourage the inclusion of the geographic predictor into SDMs, although we acknowledge some limitations of the methodology. As stated above, the geographic predictor may be a useful proxy for other variables or processes that are not included in the model, such as dispersal limitation, site fidelity or conspecific attraction [64,65]. However, this predictor is constrained to the current distribution of the species, and if the model is driven primarily by geography (as it occurs in our *space-included* models), future favourability will be more or less adjusted to the current range. In these cases, the geographic predictor will not account for the dynamic nature of the processes that is representing. However, it is worth noting that models that include the geographic variable are not always driven so strongly by this predictor [14]. Ideally, SDMs should directly incorporate variables related to the processes instead of using the geographic proxy. Some of these variables would be species’ life-history traits related to range-shift abilities, i.e., dispersal ability and also traits indicative of establishment and proliferation in new environments [73,74]. Dispersal has been used to adjust future suitable areas [75] but this solution remains problematic since this variable (or any of the traits used) is also static, as a single dispersal value for the species is incorporated. It is thus desirable to spatially incorporate intra-specific variation of the traits used, but the data required are not (and will not be in the short-term) available for most species. Additionally, dispersal behaviour can be driven by environmental factors and therefore could change in time, being even more difficult to incorporate it in models’ forecasts. Thus, until the inclusion of traits into SDMs is refined, we consider important the inclusion of geographic predictors in modelling procedures.

Models including the geographic predictor performed better than the ones excluding it. Therefore, forecasted future suitability is likely to be more precise when using those models. However, we cannot be certain of what is going to happen in the future and it is also likely that true favourable areas will be somewhere in between the two projections (with and without the geographic predictor). This is because, as illustrated by our *space-included* models, it is unlikely that future species ranges will be entirely different even if climatic conditions change, so that species distribution ranges are expected to shift gradually over the time period studied. In other words, it is doubtful that most of the current distribution of bustards in the south of Europe will disappear in less than one hundred years due exclusively to climate changes. Therefore, populations occupying current high favourability areas and their habitat should be a priority for management and conservation policies.

## Supporting Information

S1 Appendix Additional figures. (PDF)

S2 Appendix Methods, results and projections for three additional SDMs. (PDF)
