# Supplementary material for: Forecasting Large-Scale Habitat Suitability of European Bustards under Climate Change: The Role of Environmental and Geographic Variables
Source: PLoS One. 2016 Mar 3;11(3):e0149810. doi: 10.1371/journal.pone.0149810 (PMC4777476; doi:10.1371/journal.pone.0149810)

Forecasting Large-Scale Habitat Suitability of European Bustards under Climate Change: The Role of Environmental and Geographic Variables

Alba Estrada, M. Paula Delgado, Beatriz Arroyo, Juan Traba & Manuel B. Morales. Plos One.

S1 Appendix. Additional figures.

Figure A. Favourability classes for the little bustard. Black cells represent high favourable areas, white cells represent unfavourable areas, and grey cells represent intermediate favourability areas. a) Present favourability according to the *space-included* model; b) future favourability in 2080 according to the *space-included* model and the GCM HADCM3; c) present favourability according to the *space-excluded* model; d) future favourability in 2080 according to the *space-excluded* model and the GCM HADCM3.

We considered the following criterion to establish the classes: If the predicted favourability was higher than 0.8, which means that the odds are more than 4:1 favourable to the species, the cell was considered as highly favourable. Areas with a favourability value lower than 0.2 (odds less than 1:4) were considered unfavourable to the species. The remaining cells were considered as intermediate favourability areas (Muñoz et al., 2005). These thresholds distinguish areas that are clearly favourable to those that are clearly unfavourable.

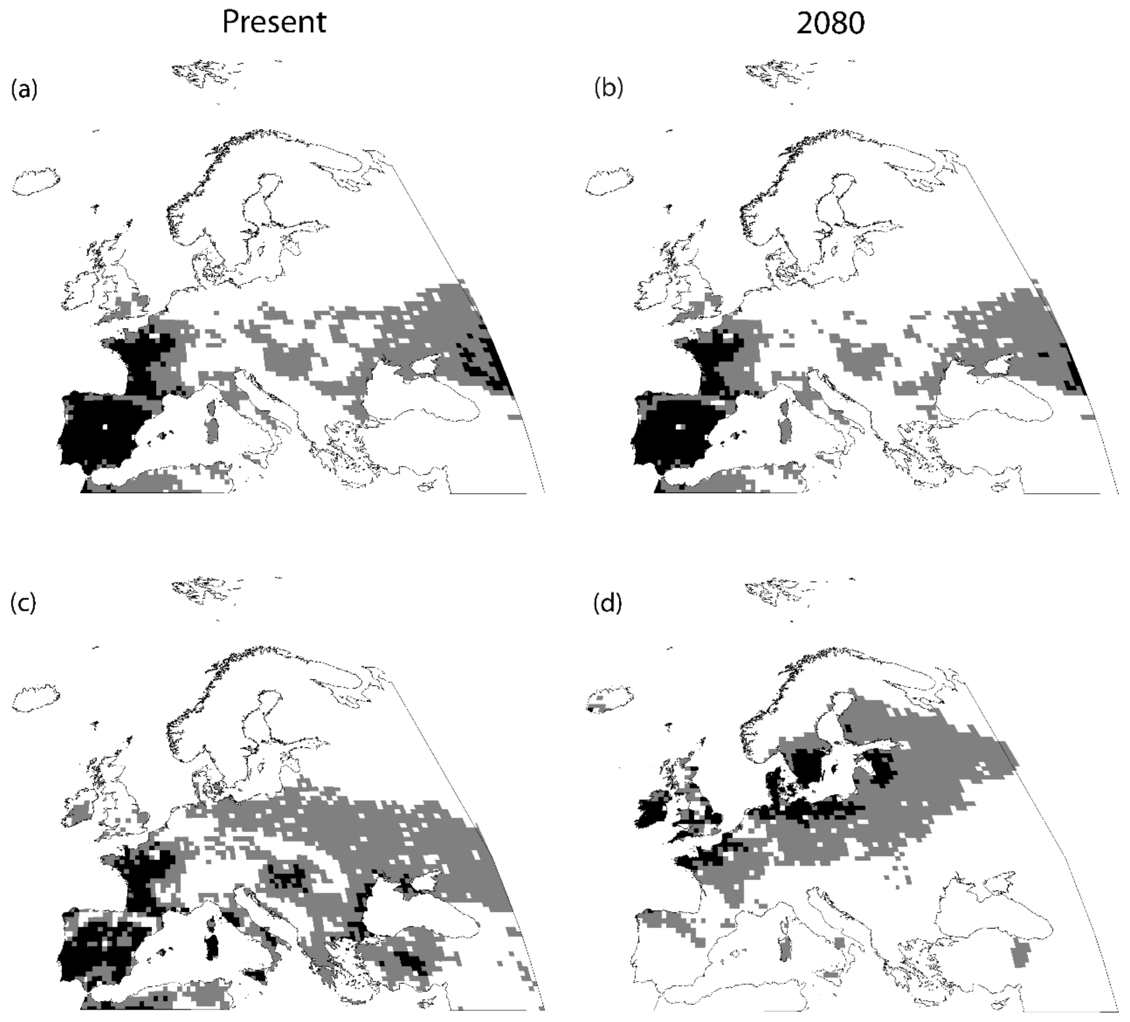

Figure B. Favourability classes for the great bustard. Black cells represent high favourable areas, white cells represent unfavourable areas, and grey cells represent intermediate favourability areas. a) Present favourability according to the *space-included* model; b) future favourability in 2080 according to the *space-included* model and the GCM HADCM3; c) present favourability according to the *space-excluded* model; d) future favourability in 2080 according to the *space-excluded* model and the GCM HADCM3. The criterion followed to establish the classes is detailed in S1 Fig.

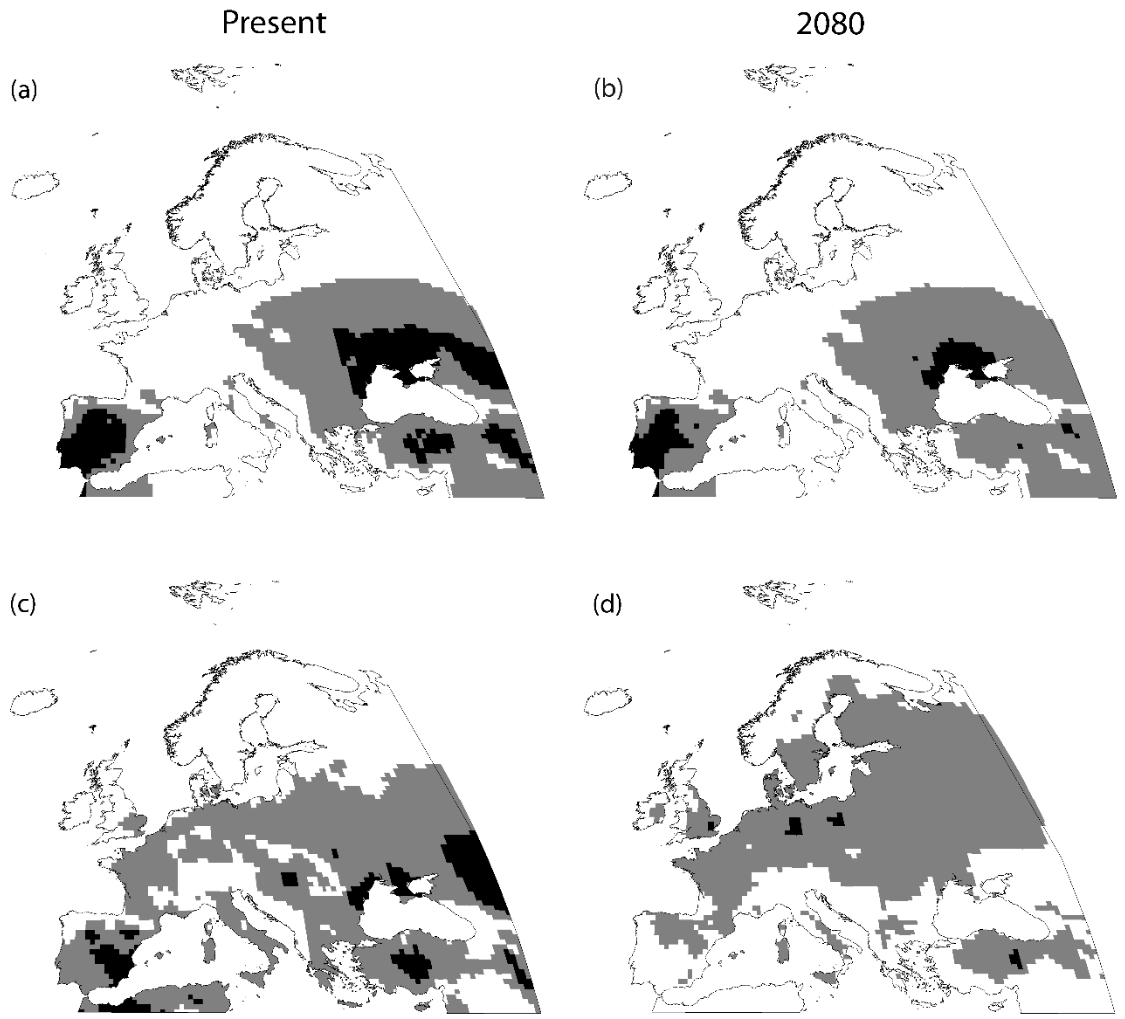

Figure C. Favourability for the little bustard in 2050 and 2080 in three GCMs according to the *space-included* model. Favourability ranges from zero (white cells) to one (black cells).

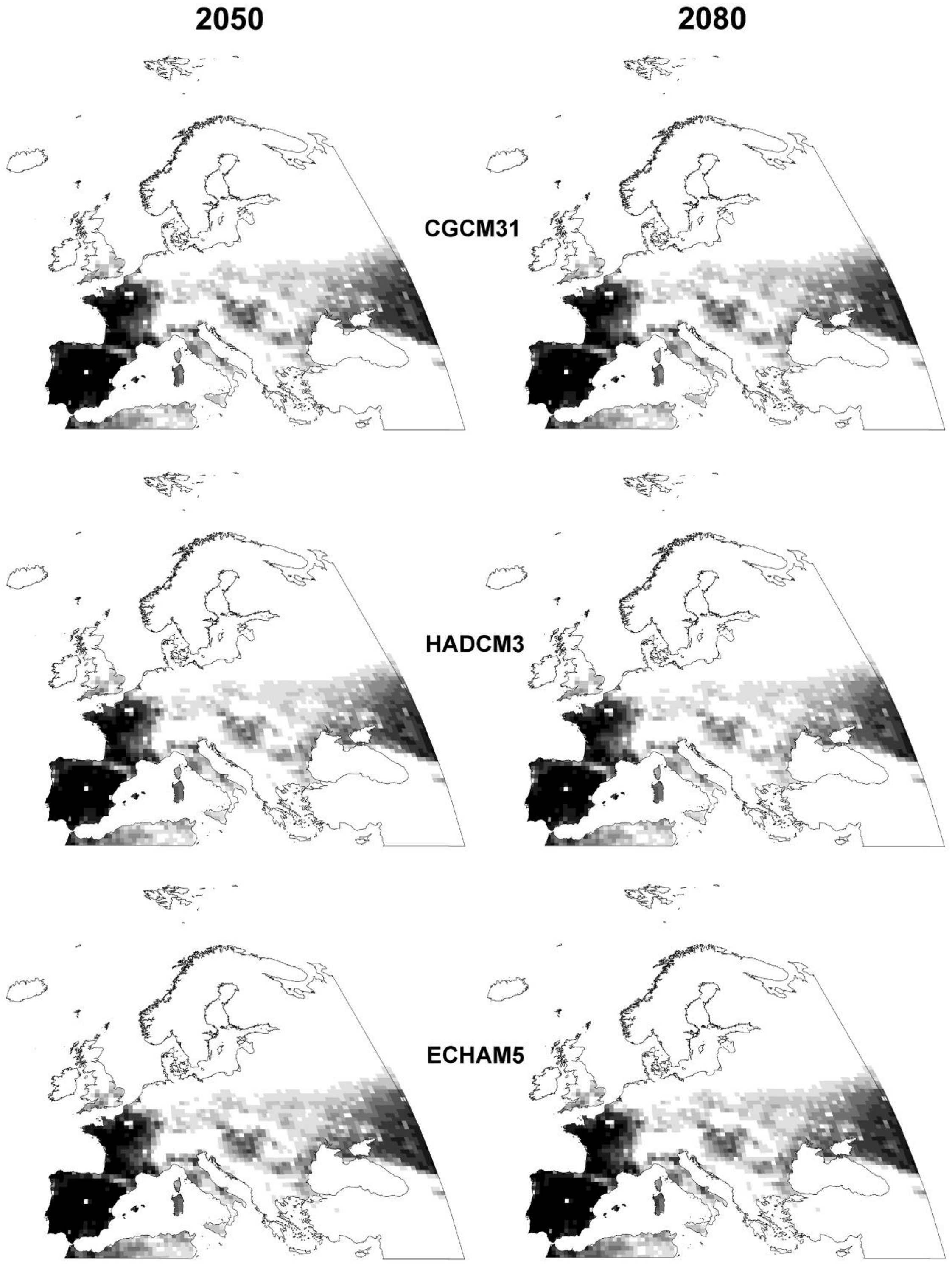

Figure D. Favourability for the little bustard in 2050 and 2080 in three GCMs according to the *space-excluded* model. Favourability ranges from zero (white cells) to one (black cells).

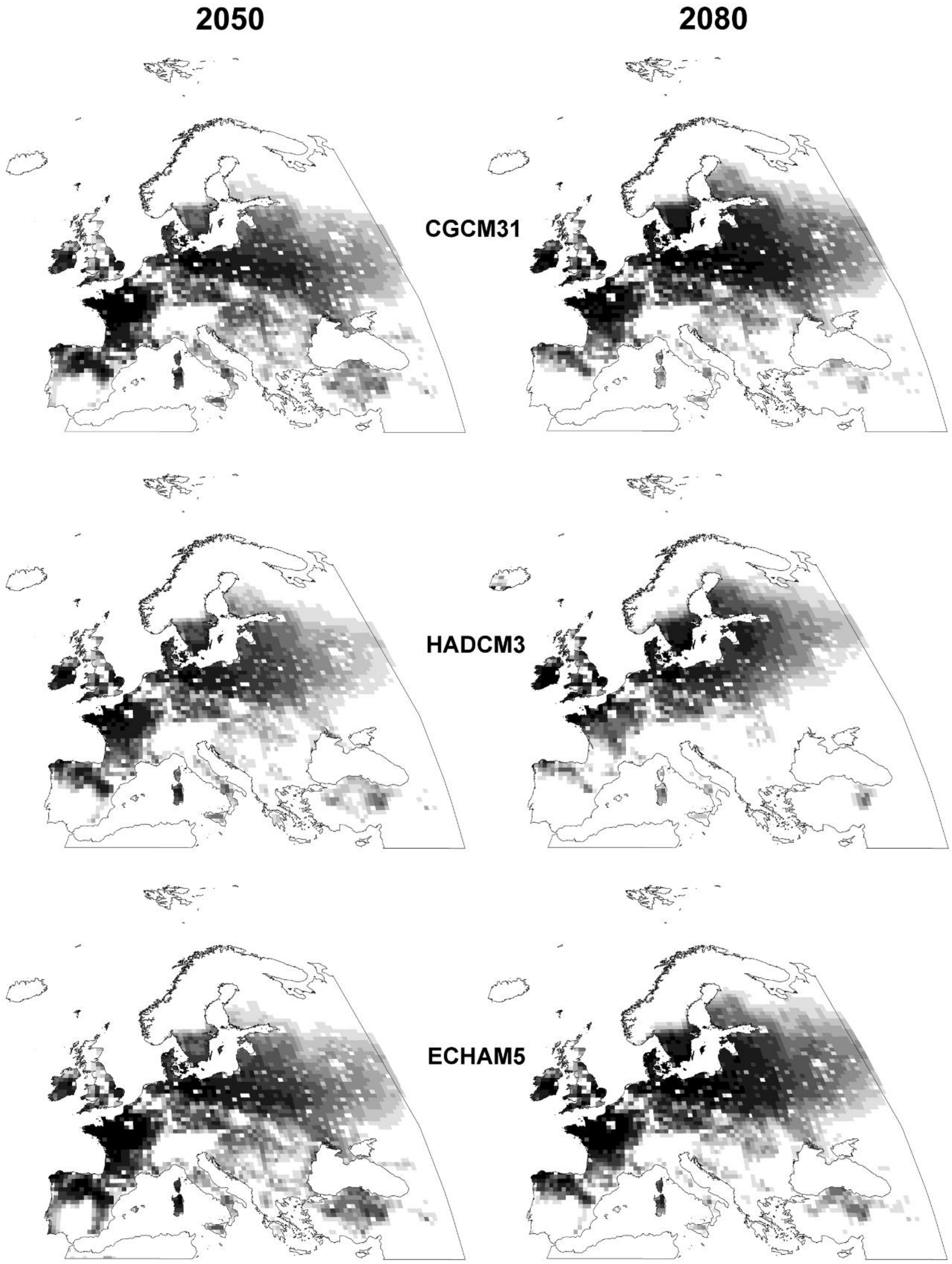

Figure E. Favourability for the great bustard in 2050 and 2080 in three GCMs according to the *space-included* model. Favourability ranges from zero (white cells) to one (black cells).

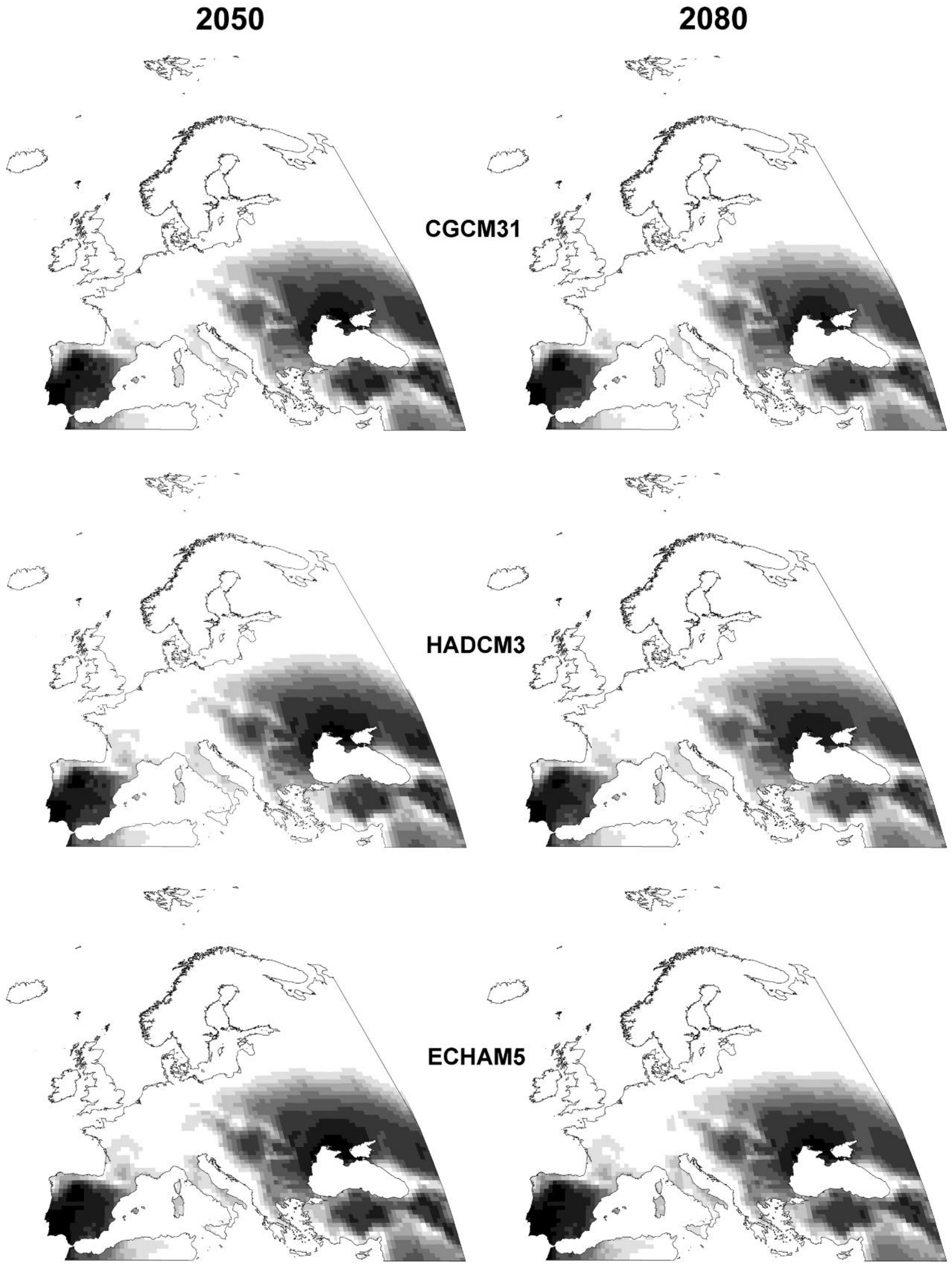

Figure F. Favourability for the great bustard in 2050 and 2080 in three GCMs according to the space-excluded model. Favourability ranges from zero (white cells) to one (black cells).

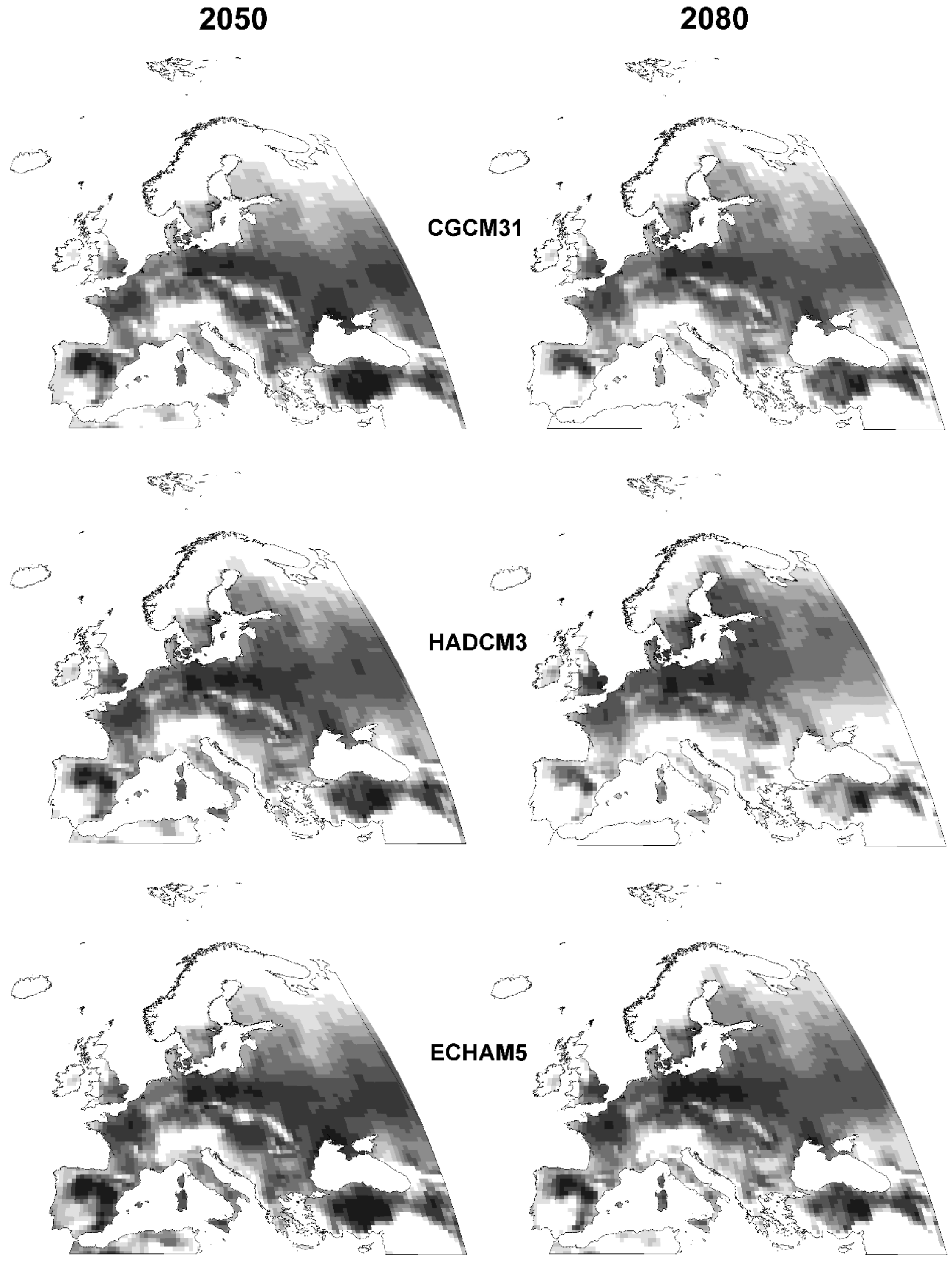

Supplement: S1 Appendix — (PDF) [file pone.0149810.s001.pdf]
