# Supplementary material for: Forecasting Large-Scale Habitat Suitability of European Bustards under Climate Change: The Role of Environmental and Geographic Variables
Source: PLoS One. 2016 Mar 3;11(3):e0149810. doi: 10.1371/journal.pone.0149810 (PMC4777476; doi:10.1371/journal.pone.0149810)
Supplement: S2 Appendix — (PDF) [file pone.0149810.s002.pdf]

Forecasting Large-Scale Habitat Suitability of European Bustards under Climate Change: The Role of Environmental and Geographic Variables

Alba Estrada, M. Paula Delgado, Beatriz Arroyo, Juan Traba & Manuel B. Morales. Plos One.

S2 Appendix. Methods, results and projections for three additional SDMs.

To ascertain that our results are generalizable for different SDMs, we also modelled the distribution of both species with three other modelling techniques widely used in species distribution modelling: Boosted regression trees (GBM) [1], Random Forest (RF) [2] and Multiple Adaptive Regression Splines (MARS) [3]. Variables used are those detailed in Table 1. In the case of geographic variables, we did not consider the trend surface variable to avoid the use of logistic regression techniques in these SDMs. Thus, we just used the independent geographic variables (X, Y, X^2, Y^2, X^3, Y^3, XY, X^2Y, XY^2), together with climatic, topographical and land-use variables. Models were performed in R [4] with the default options of the package *biomod2* [5]. We performed two types of models, one that included environmental and geographic variables (*space-included* model), and a second one that only includes environmental variables (*space-excluded* model). Models were trained on a 70% random sample of the original data and predictive accuracy was tested on the remaining 30%. The discrimination power of the models was assessed with the AUC and with the True Skill Statistic (TSS). Suitable areas were projected to 2080 according to the GCM HADCM3.

In all cases, evaluation metrics are better for the models that included geographic variables (S1 Table). Current and future projections may differ depending on the modelling technique (see figures below). However, in all cases, similar patterns are obtained: Current suitable areas are similar with and without the inclusion of geographic variables. Nonetheless, future suitable areas are less related to the current distribution of the species in the *space-excluded* models, and in the case of the little bustard future suitable areas are far away from current distribution. These areas are highly unlikely to be occupied in less than 100 years given the mentioned constraints to the species' dispersal (see main text).

Table A. Evaluation metrics of each model when they were assessed on the test dataset.

Species	Model	AUC	TSS
Little bustard	GBM *space-included*	0.969	0.815
	GBM *space-excluded*	0.960	0.797
	MARS *space-included*	0.972	0.877
	MARS *space-excluded*	0.942	0.762
	RF *space-included*	0.979	0.855
	RF *space-excluded*	0.966	0.780
Great bustard	GBM *space-included*	0.928	0.726
	GBM *space-excluded*	0.896	0.626
	MARS *space-included*	0.909	0.710
	MARS *space-excluded*	0.832	0.572
	RF *space-included*	0.958	0.852
	RF *space-excluded*	0.916	0.697

Figure A. Suitability for the little bustard at present and in 2080 according to the GBM model. a) Present suitability according to the *space-included* model; b) future suitability in 2080 according to the *space-included* model and the GCM HADCM3; c) present suitability according to the *space-excluded* model; d) future suitability in 2080 according to the *space-excluded* model and the GCM HADCM3. Suitability ranges from zero (white cells) to one (black cells).

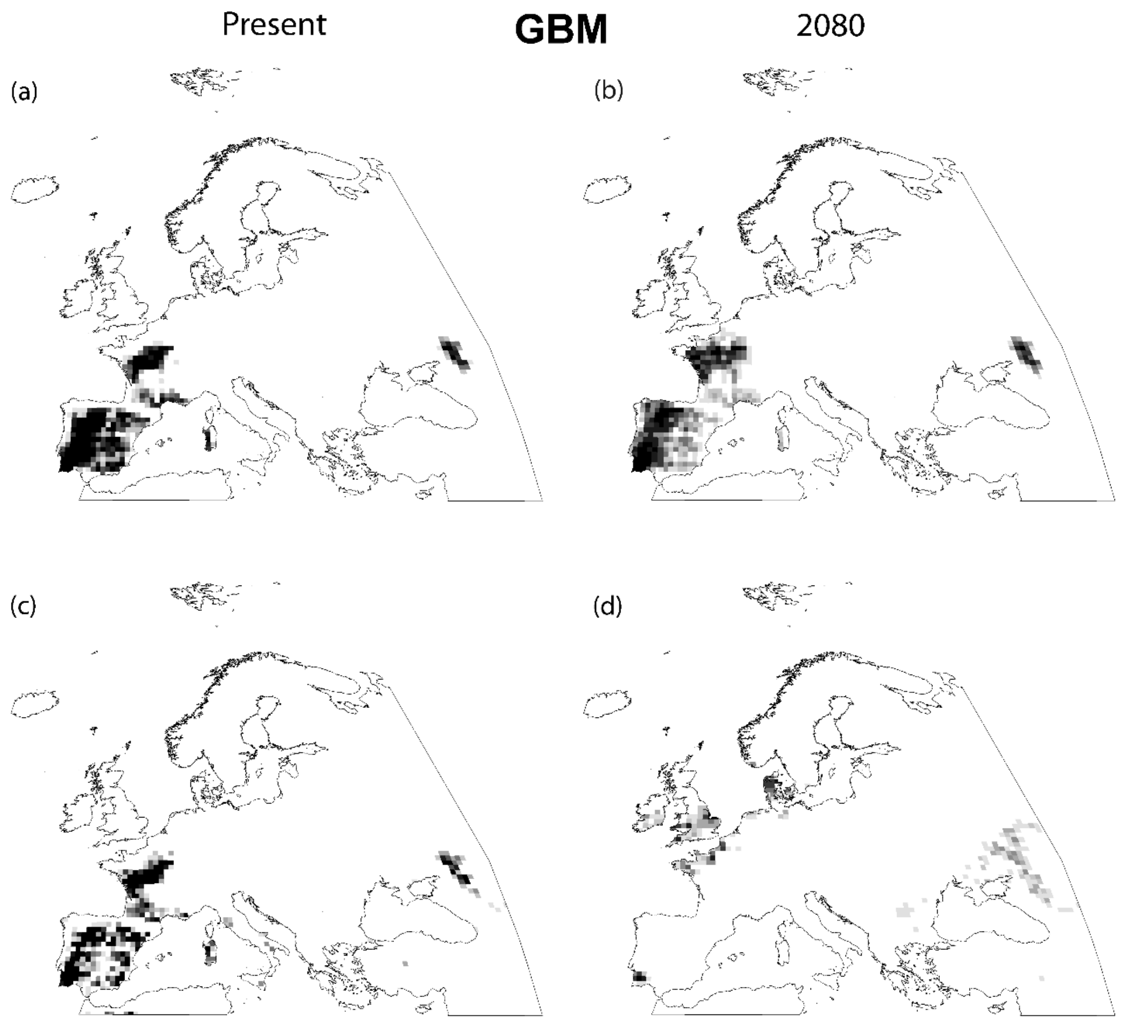

Figure B. Suitability for the little bustard at present and in 2080 according to the MARS model. a) Present suitability according to the *space-included* model; b) future suitability in 2080 according to the *space-included* model and the GCM HADCM3; c) present suitability according to the *space-excluded* model; d) future suitability in 2080 according to the *space-excluded* model and the GCM HADCM3. Suitability ranges from zero (white cells) to one (black cells).

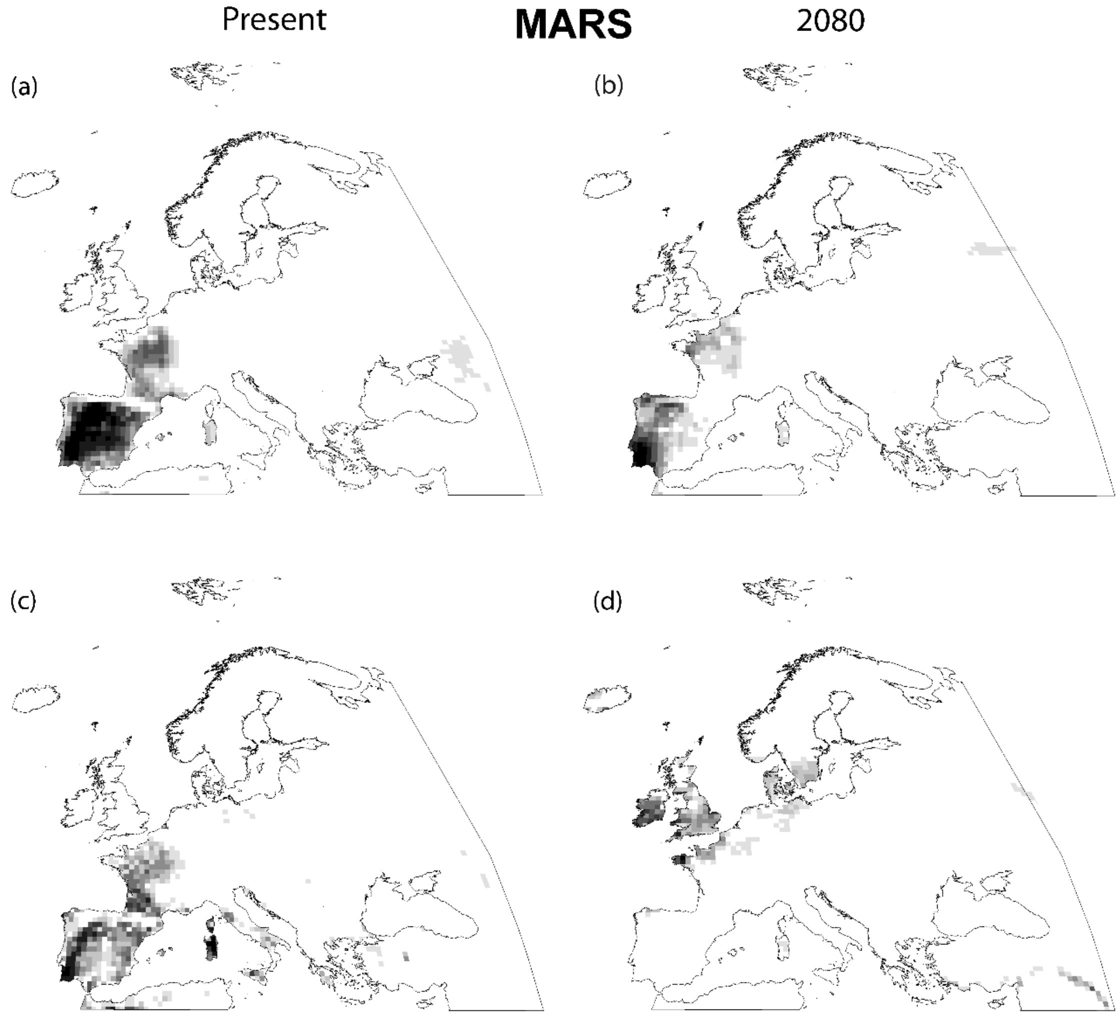

Figure C. Suitability for the little bustard at present and in 2080 according to the RF model. a) Present suitability according to the *space-included* model; b) future suitability in 2080 according to the *space-included* model and the GCM HADCM3; c) present suitability according to the *space-excluded* model; d) future suitability in 2080 according to the *space-excluded* model and the GCM HADCM3. Suitability ranges from zero (white cells) to one (black cells).

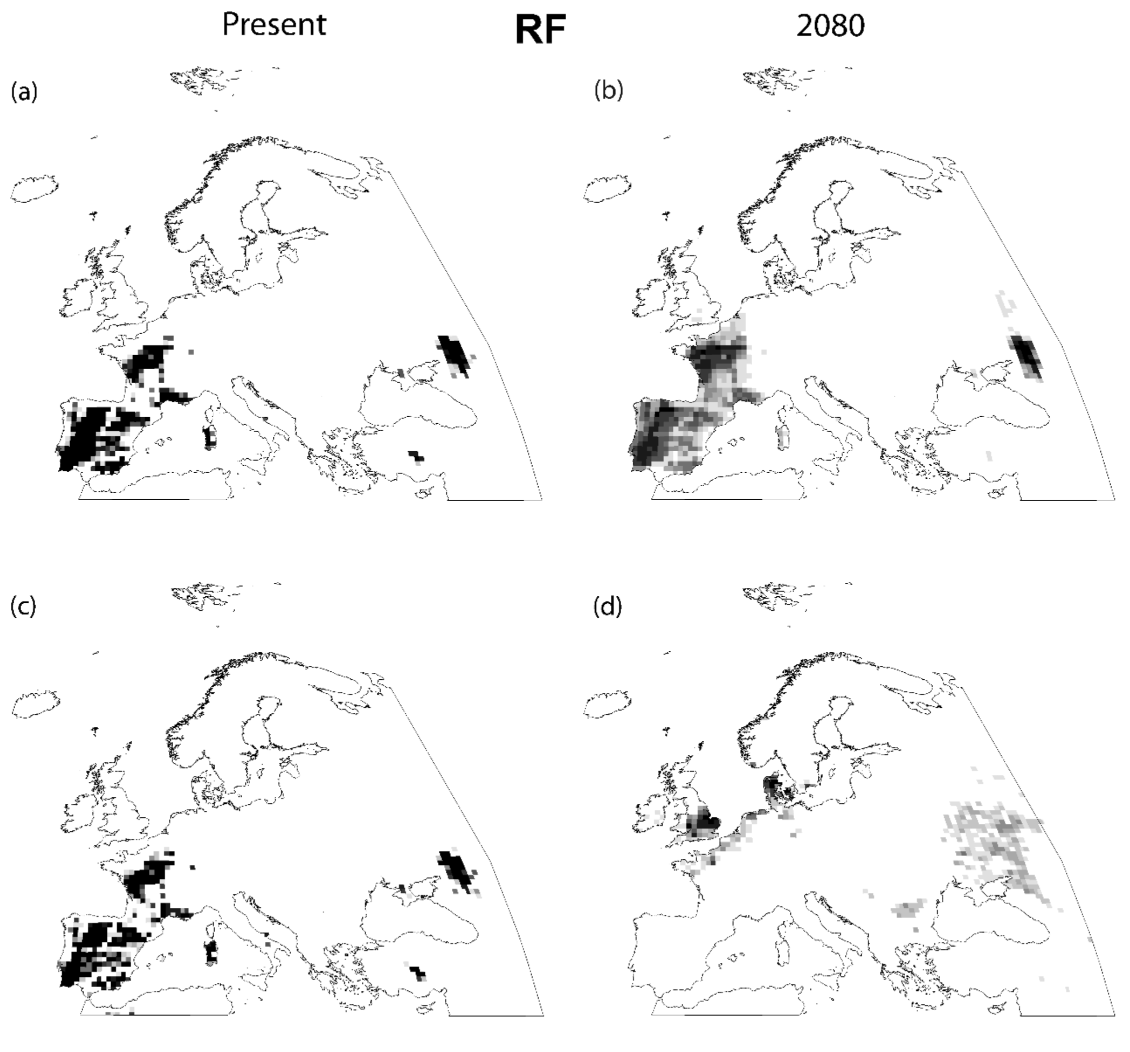

Figure D. Suitability for the great bustard at present and in 2080 according to the GBM model. a) Present suitability according to the *space-included* model; b) future suitability in 2080 according to the *space-included* model and the GCM HADCM3; c) present suitability according to the *space-excluded* model; d) future suitability in 2080 according to the *space-excluded* model and the GCM HADCM3. Suitability ranges from zero (white cells) to one (black cells).

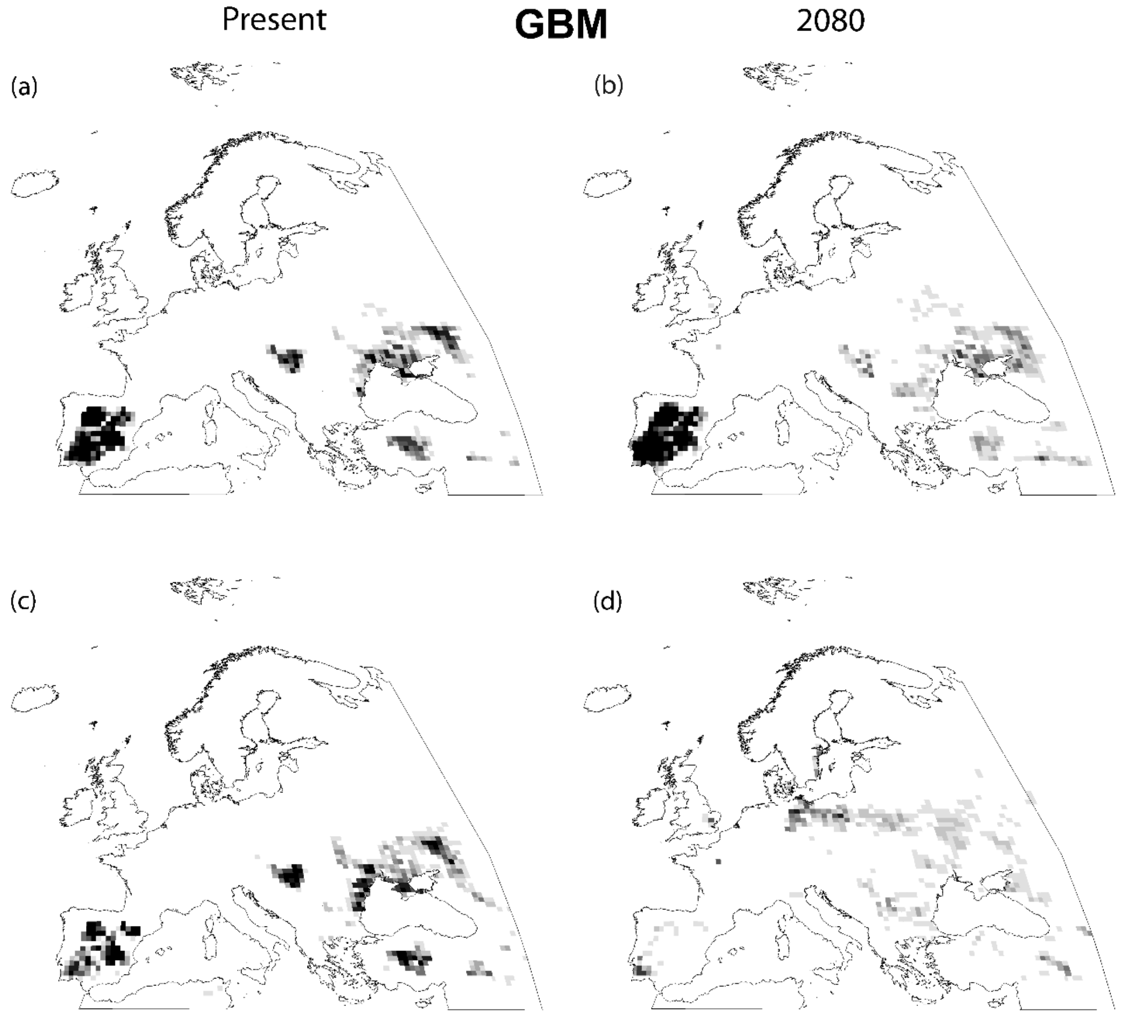

Figure E. Suitability for the great bustard at present and in 2080 according to the MARS model. a) Present suitability according to the *space-included* model; b) future suitability in 2080 according to the *space-included* model and the GCM HADCM3; c) present suitability according to the *space-excluded* model; d) future suitability in 2080 according to the *space-excluded* model and the GCM HADCM3. Suitability ranges from zero (white cells) to one (black cells).

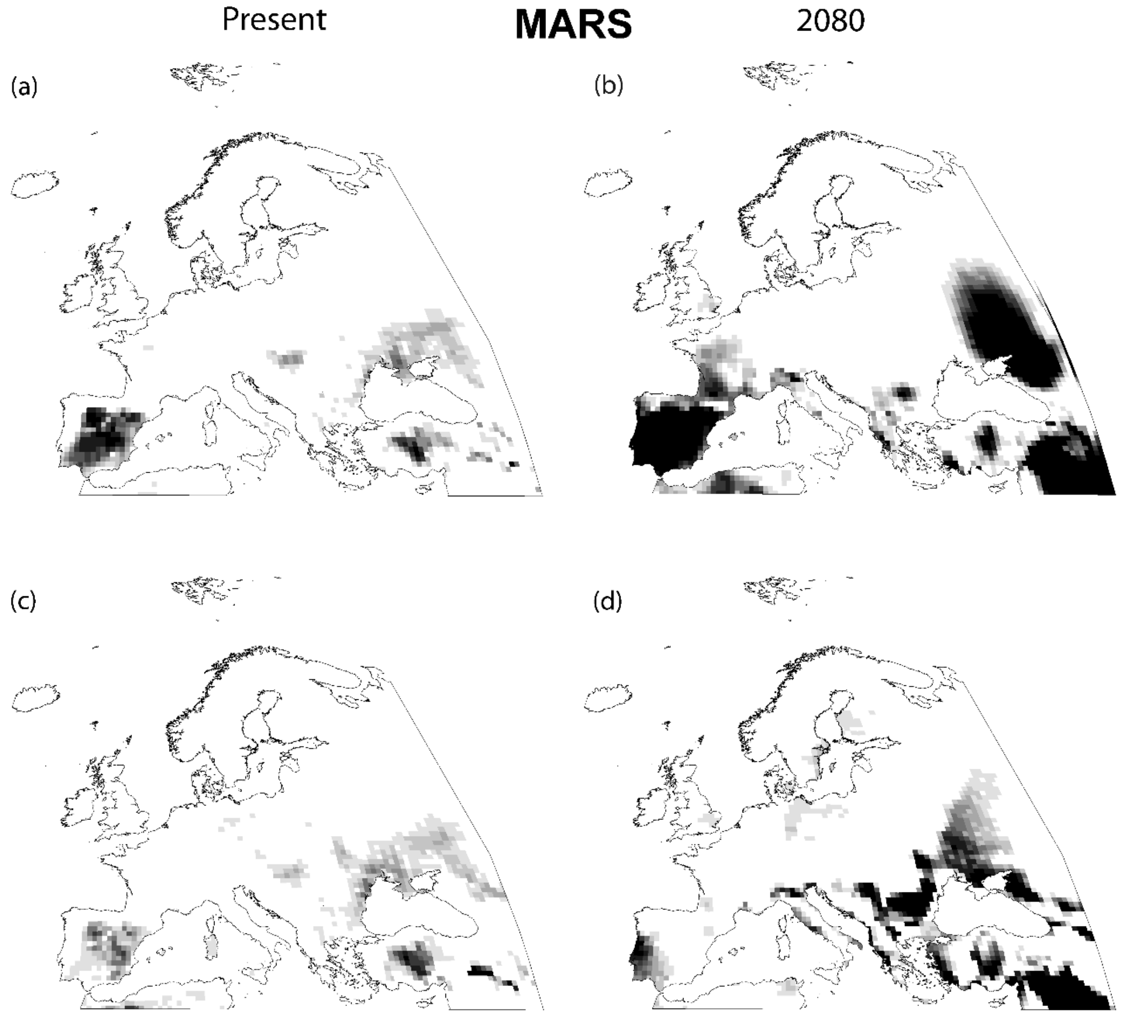

Figure F. Suitability for the great bustard at present and in 2080 according to the RF model. a) Present suitability according to the *space-included* model; b) future suitability in 2080 according to the *space-included* model and the GCM HADCM3; c) present suitability according to the *space-excluded* model; d) future suitability in 2080 according to the *space-excluded* model and the GCM HADCM3. Suitability ranges from zero (white cells) to one (black cells).

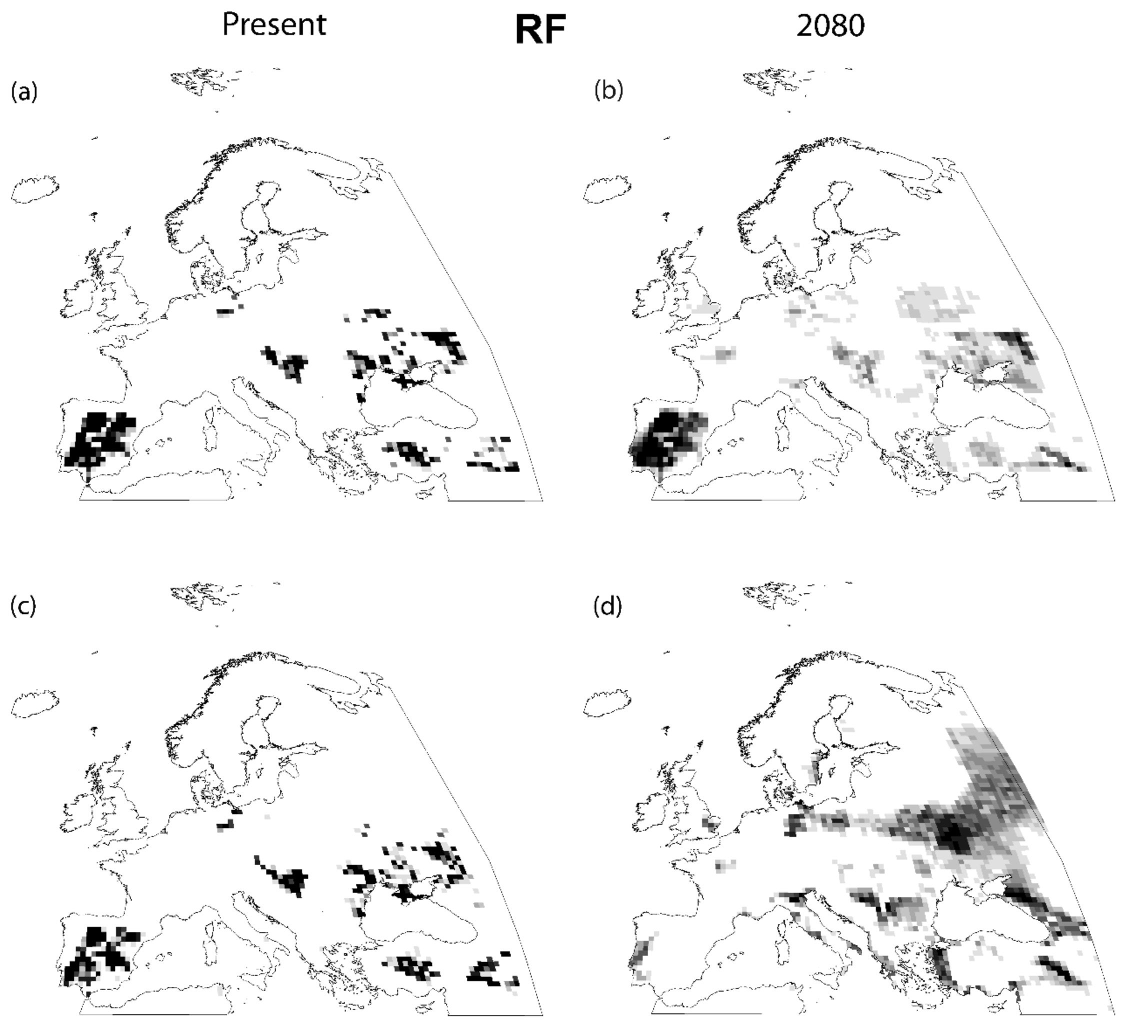

References

1. Ridgeway G. The state of boosting. Computing Science and Statistics 1999; 31: 172-181.
2. Breiman L. Random Forests. Machine Learning. 2001; 45: 5-32.
3. Friedman J. Multivariate Adaptive Regression Splines. The Annals of Statistics. 1991; 19: 1-141.
4. R Core Team. R: A language and environment for statistical computing. R Foundation for Statistical Computing, Vienna, Austria. URL http://www.R-project.org/. 2014.
5. Thuiller W, Georges D, Engler R. biomod2: Ensemble platform for species distribution modeling. R package version 3.1-25. http://CRAN.R-project.org/package=biomod2. 2013.
